# Pragmatic Considerations When Extracting DNA for Metagenomics Analyses of Clinical Samples

**DOI:** 10.3390/ijms241411262

**Published:** 2023-07-09

**Authors:** Claudio Neidhöfer, Maria Bagniceva, Nina Wetzig, Martin A. Sieber, Ralf Thiele, Marijo Parčina

**Affiliations:** 1 Institute of Medical Microbiology, Immunology and Parasitology, University Hospital Bonn, Venusberg Campus 1, 53127 Bonn, Germany; 2 Institute for Functional Gene Analytics, Bonn-Rhein-Sieg University of Applied Sciences, 53757 Sankt Augustin, Germany

**Keywords:** human microbiome, microbiome analyses, microbial community structure, DNA extraction protocols, extraction-linked bias, automation of sample processing

## Abstract

Microbiome analyses are essential for understanding microorganism composition and diversity, but interpretation is often challenging due to biological and technical variables. DNA extraction is a critical step that can significantly bias results, particularly in samples containing a high abundance of challenging-to-lyse microorganisms. Taking into consideration the distinctive microenvironments observed in different bodily locations, our study sought to assess the extent of bias introduced by suboptimal bead-beating during DNA extraction across diverse clinical sample types. The question was whether complex targeted extraction methods are always necessary for reliable taxonomic abundance estimation through amplicon sequencing or if simpler alternatives are effective for some sample types. Hence, for four different clinical sample types (stool, cervical swab, skin swab, and hospital surface swab samples), we compared the results achieved from extracting targeted manual protocols routinely used in our research lab for each sample type with automated protocols specifically not designed for that purpose. Unsurprisingly, we found that for the stool samples, manual extraction protocols with vigorous bead-beating were necessary in order to avoid erroneous taxa proportions on all investigated taxonomic levels and, in particular, false under- or overrepresentation of important genera such as *Blautia, Faecalibacterium,* and *Parabacteroides.* However, interestingly, we found that the skin and cervical swab samples had similar results with all tested protocols. Our results suggest that the level of practical automation largely depends on the expected microenvironment, with skin and cervical swabs being much easier to process than stool samples. Prudent consideration is necessary when extending the conclusions of this study to applications beyond rough estimations of taxonomic abundance.

## 1. Introduction

Understanding the composition and diversity of microbiomes in different bodily environments, such as the human gut, skin, and other body sites, as well as in the environments that surround us is critical for identifying the association between microbial communities and health and disease [1,2]. However, microbiome research is a challenging field due to the many factors that affect it. Identifying which of these factors are practically significant can be difficult. Modern NGS and metagenomic analysis methods allow clinical laboratory scientists to identify multiple infections simultaneously; a single metagenomic assay can identify rare and/or emerging pathogens and reveal the role of the microbiome in the development of human dysbiotic conditions, and infectious and chronic diseases [3]. Automation of the DNA extraction process has the potential to greatly reduce the time and labor required for microbiome analysis, making it more accessible to clinical researchers and ultimately leading to improved patient outcomes.

The accuracy and reliability of microbiome analyses are highly dependent on the quality of DNA extraction as this process is a critical step that can affect downstream analysis [4,5,6,7,8,9,10,11,12], and several DNA extraction protocols have been developed for different sample types [13,14,15,16]. Differences that were noticed in previous studies were mainly, but not only, linked to the effectiveness of DNA extraction methods in releasing DNA from microorganisms that have robust cell walls [6,7,11,17]. The identification of appropriate DNA extraction protocols is critical to ensure that microbiome analyses are reliable and accurate, particularly in a clinical setting where misidentification of microbial taxa can have serious implications for patient diagnosis and treatment. To improve the quality of research and draw more accurate conclusions, it is important to evaluate the impact of both biological and technical variables. With a focus on the unique microenvironments found in various bodily locations, our study aimed to evaluate the impact of suboptimal bead-beating during DNA extraction on diverse clinical sample types (stool, skin swabs, cervical swabs, and hospital surface swabs). The central question addressed was whether reliable taxonomic abundance estimation through amplicon sequencing requires complex targeted extraction methods in all cases, or if simpler alternatives can be effective for certain sample types to explore the possibility of automating the processing of various sample types for metagenomic analysis in clinical microbiology, which is becoming increasingly routine [18]. We hypothesized that the use of extraction protocols including more vigorous bead beating would be necessary for some sample types to avoid false underrepresentation of certain taxa, while in other cases, targeted and complex extraction methods may not provide significant advantages. 

For each clinical sample type, we included both an automated DNA extraction kit, routinely used for other diagnostic purposes in our diagnostic laboratory, and a targeted and complex manual extraction method, regularly employed in our research laboratory, specifically designed for the respective sample types under investigation. For most sample types, we even included additional available manual/automated protocols. The goal of this study was not to demonstrate that by vigorous bead-beating, actual sample composition is more reliably depicted, as this seems obvious by now, but rather to demonstrate that when a rough estimate of taxonomic abundance through amplicon sequencing is all that is needed, more practical alternatives can be an acceptable substitute for some clinical sample types.

## 2. Results

A total of 40 clinical samples were subjected to nucleic acid extraction using one or two targeted manual protocols, namely the PureLink Microbiome DNA Purification Kit (referred to as PMP kit; Thermo Fisher Scientific, Waltham, MA, USA) and the ZymoBIOMICS DNA Miniprep Kit (referred to as ZBM kit; Zymo Research Europe GmbH, Freiburg, Germany), as well as one or two automated protocols using the Maxwell 16 Tissue DNA Purification Kit (referred to as MTP kit; Promega, Mannheim, Germany) and the STARMag 96 X 4 Universal Cartridge kit (referred to as SMU kit; Seegene Inc., Seoul, Republic of Korea), which were not specifically designed for the analysis (see Section 4.2). This resulted in 120 evaluated microbiome profiles (see Table 1).

**Figure 1:**
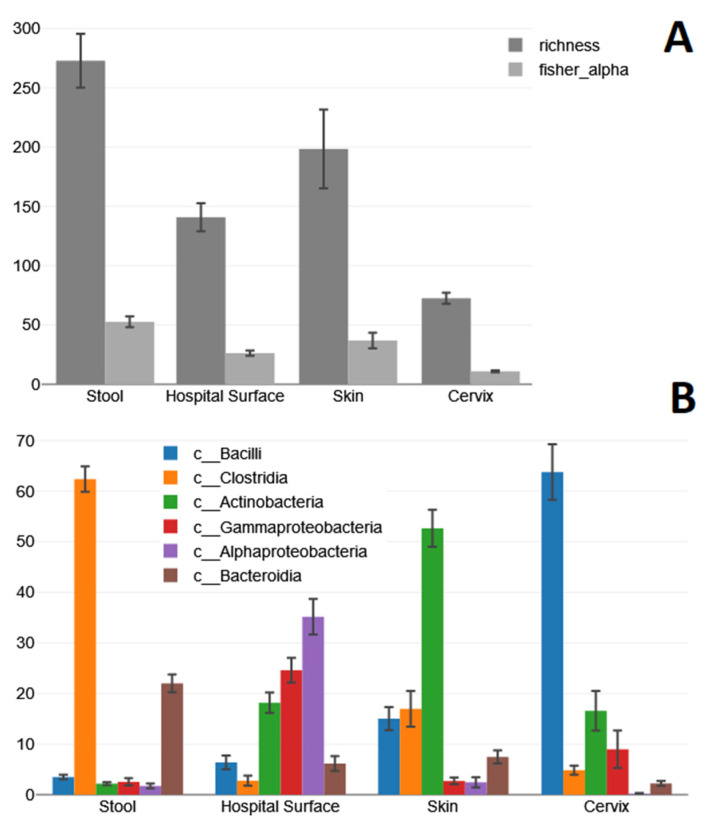
Average richness and fisher-alpha diversity for each sample type (**A**); absolute numbers on the y-axis), and mean relative abundance (y-axis) of different bacterial classes in different clinical sample types (**B**), both including standard errors. The comparison of compositions at the class level was chosen due to its ability to provide a balanced approach with reliable and abundant taxonomic resolution, as well as insights into the predictability of microorganisms’ susceptibility to lysis.

Sequencing generated a total of 11,593,429 reads with a mean read count of 96,612 reads (±45,502) per microbiome. All sequenced microbiomes passed our set minimum quality criteria of >3500 reads and >2500 merged reads. The number of reads considered non-chimeric was 1,128,382 (9.7%; 10.02% on average per sample). The OTU number varied most strongly by sample type, ranging from 30 to 626, with averages depicted in Figure 1A.

At the phylum-to-species level, all taxa with an average abundance of >0.5% were considered for the statistical analysis. These included 6 phyla, 9 classes, 25 orders, 35 families, 30 genera, and 9 species. The average richness and fisher-alpha diversity for each sample type are displayed in Figure 1A. The ANOVA showed that there were significant differences in regard to the variable richness (F = 27.53, *p* ≤ 0.001) and fisher-alpha diversity (F = 30.53, *p* ≤ 0.001). The Bonferroni post hoc test revealed that the pairwise group comparisons of Skin–Stool, Skin–Cervix, Hospital Surface–Stool, Hospital Surface–Cervix and Stool–Cervix have a *p*-value less than 0.05, both in regard to richness and fisher alpha diversity. Abundance of the six most prominent bacterial classes in different clinical sample types is displayed in Figure 1B. 

The ANOVA and post hoc tests revealed significantly more Bacilli (F = 65.41, *p* ≤ 0.001) in cervical swabs than in all other sample types (*p* < 0.05), more Clostridia (F = 225.56, *p* = ≤ 0.001) in stool than in all other samples (*p* < 0.05) and in skin than in cervical and hospital surface swabs (*p* < 0.05), more Actinobacteria (F = 37.2, *p* ≤ 0.001) in skin than in all other samples and in cervical and hospital surface swabs than in stool (*p* < 0.05), more Gamma- (F = 13.05, *p* ≤ 0.001) and Alphaproteobacteria (F = 88.32, *p* ≤ 0.001) in hospital surface swabs than in all other sample types (*p* < 0.05), and more Bacteroidia (F = 50.18, *p* ≤ 0.001) in stool than in all other samples (*p* < 0.05) and in skin than in cervical swabs (*p* < 0.05).

### 2.1. Stool Samples

Among stool samples, Bacillota represented the most commonly detected phylum (66.68%), followed by Bacteroidota (22.01%). Actinomycetota (4.63%), Pseudomonadota (4.28%), and other phyla (2.42%) were much less commonly detected. Similarly, the most commonly detected class was the Clostridia (62.37%), followed by Bacteroidia (22.01%) and Bacilli (3.49%); Gammaproteobacteria (2.55%), Coriobacteriia (2.44%), Actinobacteria (2.18%), and Alphaproteobacteria (1.73%) were much less commonly detected (see Figure 1B). We found all four of the most prominent phyla to differ significantly in abundance with different extraction protocols. The phyla Bacillota (F = 13.26, *p* < 0.001) and Actinomycetota (F = 7.75, *p* = 0.002) were more frequently detected when DNA was extracted manually, with either the ZBM or PMP kit, than with the MTP kit on the Promega Maxwell 16 (acronyms are defined in the methods section). In turn, Bacteroidota (F = 26.7, *p* < 0.001) and Pseudomonadota (F = 4, *p* = 0.03) were more frequently detected with the latter. Also, with either the ZBM or PMP kit, we detected the classes Clostridia (F = 8.87, *p* = 0.001), Bacilli (F = 5.76, *p* = 0.008), and Coriobacteriia (F = 17.19, *p* < 0.001) more frequently and the class Bacteroidia (F = 26.7, *p* < 0.001) less frequently. Among all other evaluated taxa, the orders Lachnospirales (F = 22.61, *p* < 0.001), Erysipelotrichales (F = 6.18, *p* = 0.006), Coriobacteriales (F = 17.19, *p* < 0.001), and Peptostreptococcales-Tissierellales (F = 6.22, *p* = 0.006), the familiy *Lachnospiraceae* (F = 22.11, *p* < 0.001), and the genus *Blautia* (F = 12.5, *p* < 0.001) were more frequently detected with either the ZBM or PMP kit than with the MTP kit. In microbiome profiles where DNA was extracted with the MTP kit, the order Bacteroidales (F = 26.43, *p* < 0.001), the family *Bacteroidaceae* (F = 12.67, *p* < 0.001), and the genus *Bacteroides* (F = 12.67, *p* <0.001) were instead more prevalent than in those extracted with either the ZBM or PMP kit. The genera *Faecalibacterium* (F = 4.44, *p* = 0.022) and *Parabacteroides* (F = 4.16, *p* = 0.027) were only detected significantly more frequently with the MTP kit when compared to the PMP kit.

### 2.2. Hospital Surface Swabs

Among hospital surface swabs, Pseudomonadota (59.83%) represented the most commonly detected phylum, followed by Actinomycetota (18.65%), Bacillota (9.25%), Bacteroidota (6.16%), and other phyla (6.12%). The most commonly detected classes were Alphaproteobacteria (35.16%), Gammaproteobacteria (24.6%), and Actinobacteria (18.18%). Among phyla and classes, no significant differences were found among microbiomes extracted manually with the ZBM or PMP kit and those extracted with the MTP kit on the Promega Maxwell 16. There were only one order, one family, and one genus with significant differences between extraction protocols, and this concerned a higher abundance of Staphylococcales (F = 4.41, *p* = 0.022), *Staphylococcaceae* (F = 4.52, *p* = 0.02), and *Staphylococcus* (F = 4.6, *p* = 0.019) in microbiomes extracted with the ZBM kit as compared to the MTP kit.

### 2.3. Skin Swabs

Skin swabs were characterized largely by the phyla Actinomycetota (52.95%) and Bacillota (32.05%), and to a lesser degree by Bacteroidia (7.48%) and Pseudomonadota (5.17%), whereas all other phyla only represented 2.34%. The most important classes were Actinobacteria (52.65%), Clostridia (16.97%), Bacilli (15.03%), and Bacteroidia (7.47%). In skin swabs, no significant differences were found for any considered taxa among microbiomes for which DNA was extracted manually with the PMP kit and those extracted with the MTP kit on the Promega Maxwell 16.

### 2.4. Cervical Swabs

**Figure 2:**
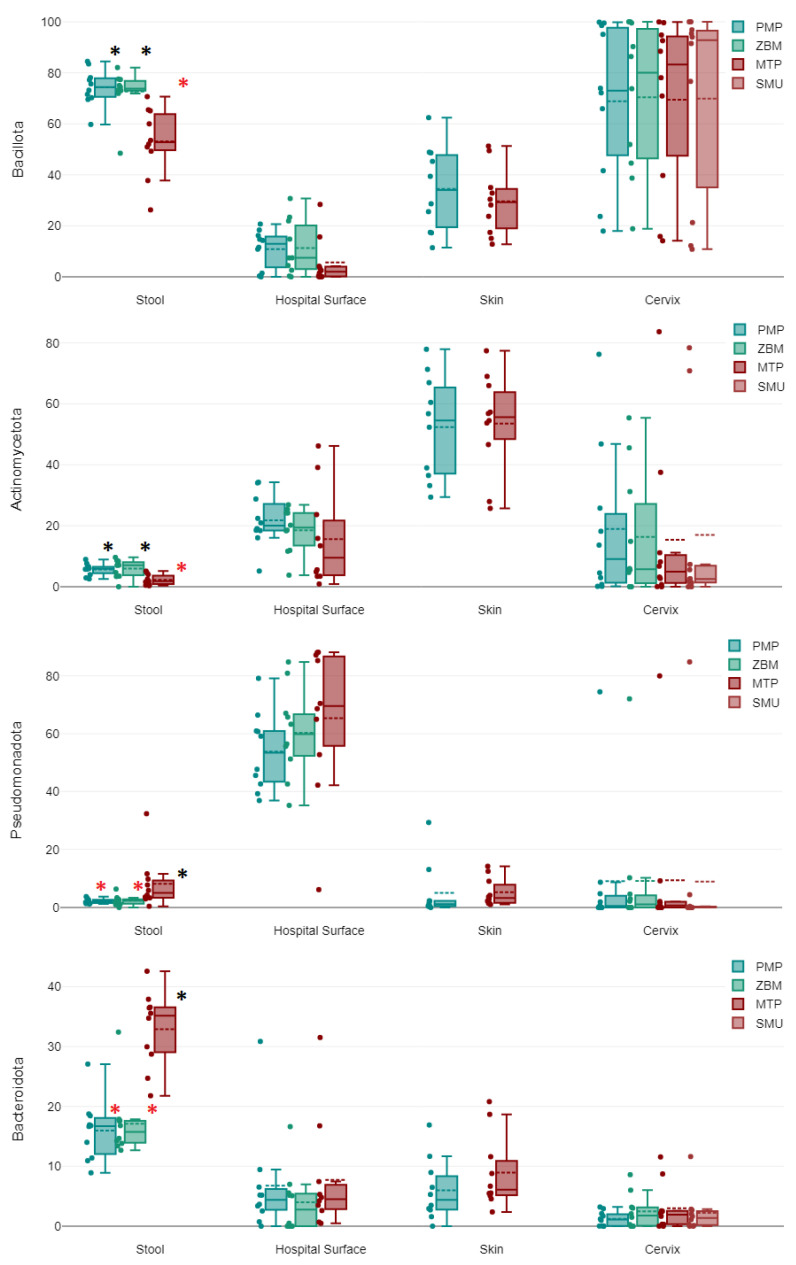
Analyzing relative abundance of bacterial phyla (y-axis) in different clinical sample types with various extraction protocols shows significant differences between extraction groups only in stool, denoted by red (lower) versus black asterisks (higher) for each phylum.

The most prominent phylum detected in cervical swabs was Bacillota (69.63%), followed by Actinomycetota (16.92%) and Pseudomonadota (9.18%). The most commonly detected class was Bacilli (63.76%), followed by Actinobacteria (16.58%), Gammaproteobacteria (8.98%), and Clostridia (4.84%). Also, in cervical swabs, no significant differences were found for any considered taxa among microbiomes for which DNA was extracted manually with the ZBM or PMP kit and those extracted with the MTP kit on the Promega Maxwell 16 or the SMU kit on the Microlab NIMBUS IVD. Figure 2 shows differences between extraction groups, which are only significant for stool samples in our study as highlighted by the asterisks.

## 3. Discussion

The results of our study indicate that the choice of DNA extraction method can have a significant impact on the composition of the microbiome in clinical sample types, but also highlights that the impact of DNA extraction methods on microbiome composition is largely dependent on the sample type being analyzed. We found significant differences in microbial community structure between manual and automated extraction methods for stool samples, but not for skin or cervical swabs. The variations observed across different sample types were not associated with the overall species richness or diversity of the specimens, but rather with the presence and abundance of certain bacterial classes with sturdy cell walls.

Previous studies examining extraction-linked bias that have looked at human stool [7,8,9], human skin [12], soil [10,11], and the intestines of koi carps [6] have found differences in microbial community structure between DNA extraction methods. Such observed discrepancies were primarily attributed to the efficacy of DNA extraction methods in liberating DNA from microorganisms that possess more sturdy cell walls such as endospores of Bacillus species [7,11,17]. We postulate that this also accounts for the higher detection of Bacillota and Actinobacteria in stool using the more elaborate manual protocols, which involve considerably more vigorous bead beating. We corroborate the important observation that the genus *Blautia*, which is certainly of great importance in gut microbiome studies unless purified by elaborate manual procedures, was hardly detectable [8]. 

In low-biomass samples, DNA extraction was reported to potentially create an even greater bias [6,17], making the introduction of positive and negative extraction controls for such samples mandatory. A study analyzing the skin microbiome found some extraction kits to have introduced bias by being majorly contaminated with bacterial DNA, but altogether, extraction did not affect microbial profiles more than inter-individual variation [12]. Some other studies that have looked at microbiomes found in human cervical swabs [19], human stool [20], and hospital swabs [21] have also found fewer differences between different extraction protocols. It seems unlikely that sample storage time had a significant effect on DNA extraction efficiency.

The presence, abundance, and prevalence of difficult-to-lyse microorganisms emerged as the primary contributing factors among the observed differences, highlighting the significance of the taxa present/expected in the samples as the most influential biological variable. Our study suggests that, for instance, skin, cervical and, to some extent, hospital swabs could be prepared for sequencing using comparatively rapid automated procedures when a rough assessment of taxonomic abundance is sufficient for the intended purpose. Even the MTP kit, only intended for DNA extraction from tissue, was not inferior to the kits specifically intended for that purpose. It is important to note that our analysis only included taxa with an average abundance of >0.5%, and it is possible that differences in extraction methods could impact rare taxa with low abundances. Also, there is still a need for studies involving larger sample sizes and studies examining microorganisms other than bacteria [22]. While microbiome analyses are currently largely batch-based due to extraction and sequencing protocols, the potential automation of certain sample types could represent a first step towards more routine-friendly processing. However, a related concern is that microorganisms possessing sturdy cell walls that might rarely be present in these types of specimens may go undetected.

## 4. Materials and Methods

### 4.1. Origin of the Samples

For this study, a total of 40 samples from cryo-storage of previous clinical studies were collected. Randomly, 10 stool samples, 10 skin swabs, 10 cervical swabs, and 10 hospital surface swabs were selected to include in approximately equal parts for samples of healthy controls as well as samples of patients with different health conditions that were, however, not found to bias the microbiome composition significantly in previously performed experiments [23]. Hospital surface swabs included only those from hospital sanitary inventory of patient rooms. Stool samples were stored in DNA/RNA shield-fecal collection tubes without beads (Zymo Research Europe GmbH, Freiburg, Germany), and all other samples were stored in 1 mL eNAT medium tubes (Copan, Brescia, Italy). Samples were cryo-stored for less than 18 months before DNA was extracted for this study. Aliquots were used for different extraction protocols.

### 4.2. DNA Extraction

DNA extraction was performed strictly according to the respective manufacturers’ instructions. From stool samples and hospital surface swabs, DNA was extracted manually with the column-based ZymoBIOMICS DNA Miniprep Kit (Zymo Research Europe GmbH, Freiburg, Germany) applicable for all investigated sample types, referred to throughout the text as ZBM kit, the column-based PureLink Microbiome DNA Purification Kit (Thermo Fisher Scientific, Waltham, MA, USA) applicable for all investigated sample types, referred to throughout the text as PMP kit, and with the automated Maxwell 16 Tissue DNA Purification Kit (Promega, Mannheim, Germany) only intended for tissue samples, referred to throughout the text as MTP kit on the Promega Maxwell 16 (Promega, Mannheim, Germany). From skin swabs, DNA was extracted manually with the PMP Kit and with the automated MTP kit on the Promega Maxwell 16 (Promega, Mannheim, Germany). From cervical swabs, DNA was extracted manually with the ZBM and PMP kits and with the automated MTP kit and the automated STARMag 96 X 4 Universal Cartridge kit (Seegene Inc., Seoul, Republic of Korea) intended for tissue samples, cells, bacteria, serum, plasma, whole blood, nasopharyngeal swabs, nasopharyngeal aspirates, bronchoalveolar lavages, urine, stool, sputum, genital swabs, and liquid-based cytology specimens, referred to throughout the text as SMU kit on the Microlab NIMBUS IVD (Seegene Inc., Seoul, Republic of Korea). Not all samples were extracted with all four extraction protocols as the aim was to test at least one automated kit not designed for the sample type with one targeted manual method routinely used in our research lab. Stool and hospital swabs are routinely extracted with either the MTP or ZBM kit, as such both were included. For cervical swabs, the SMU kit was compared as an automated kit not designed for that purpose as it is used in our lab for studying viral DNA from cervical swabs. Alongside the samples, DNA was extracted with all four extraction protocols from Microbial Community Standards (Zymo Research Europe GmbH, Freiburg, Germany). No negative control was included in our study focusing on taxa with abundances exceeding 0.5% as minor kit contaminants do not reach such levels in non-sterile clinical samples, and the results do not suggest interference or the necessity for a negative control.

### 4.3. Library Preparation and Sequencing

The 16S rRNA gene sequencing libraries were constructed using the Quick-16S NGS Library Prep Kit (Zymo Research Europe GmbH, Freiburg, Germany) with its included optimized V1-V2 primer pair in runs that included 94 samples, the the positive control, and a negative control. Modified V1-V2 primer pairs were chosen for the analysis due to their advantages in genus-level resolution, length, and ability to detect certain potentially pathogenic genera [23,24,25]. After pooling, the DNA was quantified with the QuantiFluor dsDNA System on the Quantus Fluorometer (Promega GmbH, Walldorf, Germany) and diluted strictly according to the Illumina protocol for MiSeq sample preparation. For the final library, a loading concentration of 10 pM was chosen and a 10% Illumina v3 PhiX spike-in control was added before running it on the Illumina MiSeq platform with the 500 cycle v2 Illumina MiSeq Reagent Kit. All reagents and equipment for sequencing samples were obtained from Illumina, San Diego, CA, USA. Raw reads have been uploaded to the European Nucleotide Archive; accession PRJEB60950, ERA21159872.

### 4.4. Bioinformatic Analysis

The bioinformatics analysis included three main parts, starting with the preprocessing of raw pair-end reads. Following preprocessing, the sequences were assigned to taxonomies. Finally, a statistical and graphical evaluation was performed on the resulting taxa. QIIME2 (2022.8) [26] was used for both preprocessing and classification of the data. With the plugin tool DADA2 (2022.8.0) [27], forward and reverse reads were trimmed from the 3′ end at position 249, while shorter reads and low-quality reads were discarded. DADA2 was also used to perform error correction, the merging of forward and reverse reads if there was an overlap of at least 12 base pairs, and chimera removal. The processed sequences were clustered into operational taxonomic units (OTUs) of 100% sequence identity and assigned to taxa using a classifier trained on full-length sequences of SILVA bacterial reference database [28] (138.1). The trained classifier was provided by QIIME2 using scikit-learn 0.24.1 and the plugin tool q2-feature-classifier [29,30]. Based on the quantified OTUs and taxa, different diversity indices were calculated using Python and the skbio.diversity library: the richness and Fisher indices as a measurement for alpha diversity. Microbial richness was determined by quantifying the number of unique operational taxonomic units. Unclassified taxa were included in the calculation of alpha diversity.

### 4.5. Statistical Analysis

Statistical analyses were performed using Datatab [31] version 1.12.1 for taxa frequency comparisons and with different DNA-extraction protocols; *p* values less than 0.05 were considered statistically significant. When comparing taxa abundance between two groups, first, variance equality in the samples was determined with a Levene-Test before performing a *t*-test for dependent or independent samples. When comparing taxa abundance between three or more groups ANOVA and Bonferroni Post hoc tests were performed and results only reported if also pairwise group was statistically significant. As the study design was throughout limited to a rough taxonomic abundance estimation, considering taxa with lower than 0.5% average abundance would not have made sense in particular, given that calculating statistics on low abundance taxa when looking at 10 samples of each type would have been out of place.

All data relevant to this study are included in this article or uploaded as Supplementary Information.

The University Hospital Bonn Ethics Committee confirmed that ethical approval was not required for this study since it utilized residual samples from terminated clinical and non-clinical research studies with prior consent obtained from the donors for the scientific use of the sample material.

## 5. Conclusions

When extracting DNA from skin and cervical swabs for a rough assessment of taxonomic abundance by amplicon sequencing, complex targeted extraction methods might not offer significant advantages over automated DNA extraction kits, even such, that were not originally designed for the specific sample types investigated. Processing of such sample types can hence be more easily automated than stool samples, where a more vigorous bead-beating approach generally leads to better results. Hospital swabs fell somewhere in between, with only skin bacteria not detected when samples were not extracted with vigorous bead beating. These findings are particularly relevant for laboratories planning to automate NGS analyses of clinical samples. One drawback of such automation is the possibility that it may not detect low-abundance or unexpected taxa, and this should be addressed in studies with larger sample sizes.

## Figures and Tables

**Table 1:** Sample Information and Processing Details.

Sample Type	Amount	Storage Medium	PMP	MTP	ZBM	SMU	Total
Stool samples	10	DNA/RNA Shield	yes	yes	yes		30
Hospital swabs	10	eNAT	yes	yes	yes		30
Skin swabs	10	eNAT	yes	yes			20
Cervical swabs	10	eNAT	yes	yes	yes	yes	40

## Data Availability

All data relevant to this study are included in this article or the Supplementary Information.

## Supplementary Materials

The following supporting information can be downloaded at: https://www.mdpi.com/article/10.3390/ijms241411262/s1. All data relevant to the study are included in this article. Raw reads have been uploaded to the European Nucleotide Archive; accession PRJEB60950, ERA21159872.

## References

[1] Liwinski T., Leshem A., Elinav E. (2021). Breakthroughs and bottlenecks in microbiome research. Trends Mol. Med..

[2] Gilbert J.A., Blaser M.J., Caporaso J.G., Jansson J.K., Lynch S.V., Knight R. (2018). Current understanding of the human microbiome. Nat. Med..

[3] Miller R.R., Montoya V., Gardy J.L., Patrick D.M., Tang P. (2013). Metagenomics for pathogen detection in public health. Genome Med..

[4] Bharti R., Grimm D.G. (2021). Current challenges and best-practice protocols for microbiome analysis. Brief. Bioinform..

[5] Poussin C., Sierro N., Boué S., Battey J., Scotti E., Belcastro V., Peitsch M.C., Ivanov N.V., Hoeng J. (2018). Interrogating the microbiome: Experimental and computational considerations in support of study reproducibility. Drug Discov. Today.

[6] Han Z., Sun J., Lv A., Wang A. (2019). Biases from different DNA extraction methods in intestine microbiome research based on 16S rDNA sequencing: A case in the koi carp, Cyprinus carpio var. Koi. MicrobiologyOpen.

[7] Videnska P., Smerkova K., Zwinsova B., Popovici V., Micenkova L., Sedlar K., Budinska E. (2019). Stool sampling and DNA isolation kits affect DNA quality and bacterial composition following 16S rRNA gene sequencing using MiSeq Illumina platform. Sci. Rep..

[8] Maukonen J., Simões C., Saarela M. (2012). The currently used commercial DNA-extraction methods give different results of clostridial and actinobacterial populations derived from human fecal samples. FEMS Microbiol. Ecol..

[9] MacKenzie B.W., Waite D., Taylor M.W. (2015). Evaluating variation in human gut microbiota profiles due to DNA extraction method and inter-subject differences. Front. Microbiol..

[10] Carrigg C., Rice O., Kavanagh S., Collins G., O’flaherty V. (2007). DNA extraction method affects microbial community profiles from soils and sediment. Appl. Microbiol. Biotechnol..

[11] Kuske C.R., Banton K.L., Adorada D.L., Stark P.C., Hill K.K., Jackson P.J. (1998). Small-scale DNA sample preparation method for field PCR detection of microbial cells and spores in soil. Appl. Environ. Microbiol..

[12] Bjerre R.D., Hugerth L.W., Boulund F., Seifert M., Johansen J.D., Engstrand L. (2019). Effects of sampling strategy and DNA extraction on human skin microbiome investigations. Sci. Rep..

[13] Wu W.-K., Chen C.-C., Panyod S., Chen R.-A., Wu M.-S., Sheen L.-Y., Chang S.-C. (2019). Optimization of fecal sample processing for microbiome study—The journey from bathroom to bench. J. Formos. Med. Assoc..

[14] Lear G., Dickie I., Banks J., Boyer S., Buckley H., Buckley T., Cruickshank R., Dopheide A., Handley K., Hermans S. (2018). Methods for the extraction, storage, amplification and sequencing of DNA from environmental samples. N. Z. J. Ecol..

[15] Marotz C.A., Sanders J.G., Zuniga C., Zaramela L.S., Knight R., Zengler K. (2018). Improving saliva shotgun metagenomics by chemical host DNA depletion. Microbiome.

[16] Kim D., Hofstaedter C.E., Zhao C., Mattei L., Tanes C., Clarke E., Lauder A., Sherrill-Mix S., Chehoud C., Kelsen J. (2017). Optimizing methods and dodging pitfalls in microbiome research. Microbiome.

[17] Nearing J.T., Comeau A.M., Langille M.G.I. (2021). Identifying biases and their potential solutions in human microbiome studies. Microbiome.

[18] Watts G.S., Hurwitz B.L. (2020). Metagenomic next-generation sequencing in clinical microbiology. Clin. Microbiol. Newsl..

[19] Shibata T., Nakagawa M., Coleman H.N., Owens S.M., Greenfield W.W., Sasagawa T., Ii M.S.R. (2021). Evaluation of DNA extraction protocols from liquid-based cytology specimens for studying cervical microbiota. PLoS ONE.

[20] Davis A., Kohler C., Alsallaq R., Hayden R., Maron G., Margolis E., Gutaker R.M., Reiter E., Furtwängler A., Schuenemann V.J. (2019). Improved yield and accuracy for DNA extraction in microbiome studies with variation in microbial biomass. Biotechniques.

[21] Neidhöfer C., Sib E., Benhsain A.-H., Mutschnik-Raab C., Schwabe A., Wollkopf A., Wetzig N., Sieber M.A., Thiele R., Döhla M. (2023). Examining Different Analysis Protocols Targeting Hospital Sanitary Facility Microbiomes. Microorganisms.

[22] Greathouse K.L., Sinha R., Vogtmann E. (2019). DNA extraction for human microbiome studies: The issue of standardization. Genome Biol..

[23] Condic M., Neidhöfer C., Ralser D.J., Wetzig N., Thiele R., Sieber M., Otten L.A., Warwas L.K., Hoerauf A., Mustea A. (2023). Analysis of the cervical microbiome in women from the German national cervical cancer screening program. J. Cancer Res. Clin. Oncol..

[24] Frank J.A., Reich C.I., Sharma S., Weisbaum J.S., Wilson B.A., Olsen G.J. (2008). Critical evaluation of two primers commonly used for amplification of bacterial 16S rRNA genes. Appl. Environ. Microbiol..

[25] Zhang Q., Zhang L., Wang Y., Zhao M., Chen R., Tao Z., Lyu T., Huang Z., Liao Q. (2019). An optimized 16S rRNA sequencing protocol for vaginal microbiome to avoid biased abundance estimation. Biorxiv.

[26] Bokulich N.A., Kaehler B.D., Rideout J.R., Dillon M., Bolyen E., Knight R., Huttley G.A., Gregory Caporaso J. (2018). Optimizing taxonomic classification of marker-gene amplicon sequences with QIIME 2′s q2-feature-classifier plugin. Microbiome.

[27] Bolyen E., Rideout J.R., Dillon M.R., Bokulich N.A., Abnet C.C., Al-Ghalith G.A., Alexander H., Alm E.J., Arumugam M., Asnicar F. (2019). Reproducible, interactive, scalable and extensible microbiome data science using QIIME 2. Nat. Biotechnol..

[28] Callahan B.J., McMurdie P.J., Rosen M.J., Han A.W., Johnson A.J.A., Holmes S.P. (2016). DADA2: High-resolution sample inference from Illumina amplicon data. Nat. Methods.

[29] Quast C., Pruesse E., Yilmaz P., Gerken J., Schweer T., Yarza P., Peplies J., Glöckner F.O. (2013). The SILVA ribosomal RNA gene database project: Improved data processing and web-based tools. Nucleic Acids Res..

[30] Robeson M.S., O’Rourke D.R., Kaehler B.D., Ziemski M., Dillon M.R., Foster J.T., Bokulich N.A. (2021). RESCRIPt: Reproducible sequence taxonomy reference database management. PLoS Comput. Biol..

[31] DATAtab Team (2023). DATAtab: Online Statistics Calculator.

