# Supplementary material for: Pragmatic Considerations When Extracting DNA for Metagenomics Analyses of Clinical Samples
Source: Int J Mol Sci. 2023 Jul 9;24(14):11262. doi: 10.3390/ijms241411262 (PMC10379426; doi:10.3390/ijms241411262)
Supplement: Supplementary file 1 [file ijms-24-11262-s001.zip › ijms-2480057-supplementary.pdf]

Supplementary Information

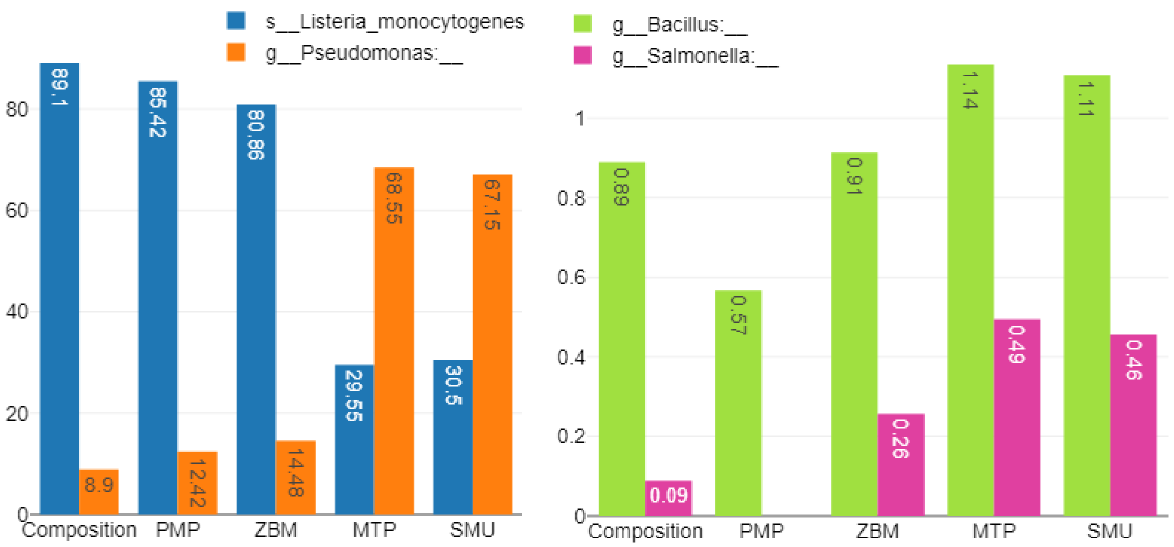

Figure S1. The sequencing results of the positive control highlight a crucial distinction between the extraction protocols: the automated ones struggle to consistently detect Gram-positive pathogens with resilient cell walls, most likely due to the significantly reduced bead-beating intensity. On the y-axis: relative prevalence.
